# Reviewer acknowledgement 2013

**DOI:** 10.1186/1471-2431-14-7

**Published:** 2014-01-16

**Authors:** Peter O'Donovan

**Affiliations:** 1 BioMed Central, 236 Gray’s Inn Road, London, WC1X 8HB, UK

## Abstract

**Contributing reviewers:**

The editors of BMC Pediatrics would like to thank all our reviewers who have contributed to the journal in Volume 13 (2013).

## 

Arturo Abdelnour

Costa Rica

Alaa Abd-Elsayed

Egypt

Abu Saleh Abdullah

USA

Saifuldeen Abdulmajeed

Iraq

Sanou Aboubakary

Canada

M Jennifer Abuzzahab

USA

Amos Olufemi Adeleye

Nigeria

M Adgent

USA

Gilbert Adimora

Nigeria

Zubair Aghai

USA

Kingsley Agho

Australia

Stewart Agras

USA

Mehmet Akman

Turkey

Nemeh Al-Akour

Jordan

Thomas Alderliesten

Netherlands

Dominik Alexander

USA

Otieno Alfred

Kenya

Sylvester Alikah

Nigeria

Bernt Alm

Sweden

Maria Fernanda Almeida

Brazil

Harith Al-Qazaz

Malaysia

Alan Altraja

Estonia

Maxwell Anah

Nigeria

Richard Anderson

USA

Susana Andrés

Spain

Odysseas Androutsos

Greece

Satinder Aneja

India

Michael Antunes

USA

Christian Apfelbacher

Germany

Nigel Armfield

Australia

Daniel Armstrong

USA

Susan Astley

USA

Elizabeth Asztalos

Canada

Leslie Atkinson

Canada

Biasini Augusto

Italy

Nicola Austin

New Zealand

Topun Austin

UK

Ambrose Awogu

Nigeria

Inge Axelsson

Sweden

Khalid Aziz

Canada

Raffaele Badolato

Italy

Paluku Bahwere

Malawi

Gianluca Baio

UK

Maria Elisabetta Baldassarre

Italy

Marilyn Ballantyne

Canada

Quentin Ballouhey

France

Karin Bammann

Germany

Srdan Banac

Croatia

Elizabeth Baranowski

UK

Egidio Barbi

Italy

Yechiel Barilan

Israel

Pierre Barker

USA

Jane Barlow

UK

Cristina Barroso

USA

Debra Bartley

Canada

Michelle Barton-Forbes

Canada

Diego Garcia Bassani

Canada

Sriparna Basu

India

Jeffrey Bauer

USA

Heiko Becher

Germany

Roberto Bellu'

Italy

Raymond Belonwu

Nigeria

David Bending

UK

Nelson Bennett

USA

Roberta Berard

Canada

Mary Berry

New Zealand

Marcel Betsch

USA

Corrado Betterle

Italy

Julie Bettinger

Canada

Andreas Beyerlein

Germany

Nita Bhandari

India

Jatinder Bhatia

USA

Shinjini Bhatnagar

India

Sibhatu Biadgilign

Ethiopia

Federico Biagi

Italy

Sebastiano Bianca

Jordan

Paolo Biban

Italy

Daniel Bingham

UK

Mona Bjelland

Norway

Piers Blackett

USA

Jonathan Blau

USA

Denise Bohrer

Brazil

Maria Bonsignore

Italy

Samudragupta Bora

USA

Jérémie Botton

France

Pascal Bovet

Switzerland

Gregory Boyle

Australia

Barbara Braden

UK

Christina Breidenassel

Germany

Alex Brito

USA

Jason Brophy

Canada

Oliviero Bruni

Italy

Mariana Brussoni

Canada

Dominique Ma Bullens

Belgium

Silvia Buratti

Italy

Scott Burgess

Australia

Jane Burns

USA

Gianfranco Butera

Italy

Stephen Butterfield

USA

Abbey Byrne

Australia

Giovanni Battista Calabri

Italy

Patrina Caldwell

Australia

Claudia Calogero

Italy

Rosa Calvo

Spain

Don Cameron

Australia

Lucia Campos Pellanda

Brazil

Franco Carnevale

Canada

Genny Carrillo Zuniga

USA

Christopher Carroll

USA

Wellington Carvalho

Brazil

Albert Catala

Spain

Carlo Catassi

Italy

Anthony Catto-Smith

Australia

Mauro Ceccanti

Italy

Mustafa Celik

Turkey

Claudia Celletti

Italy

María Florencia Cesani

Argentina

Carolina Chamorro-Vina

Canada

Ruth Chan

Hong Kong

Rima Chedid

Lebanon

Chia-Ling Chen

Taiwan

Robert Chen

USA

Aaron Chidekel

USA

Josephat Chinawa

Nigeria

Byung-Ho Choe

South Korea

Rahul Chopra

India

Robert Christensen

USA

George Chrousos

Greece

Emil Chuang

Switzerland

Manyike Chuka

Nigeria

Ndubuisi Chukwudi

Nigeria

Paige Church

Canada

Daniella Chusyd

USA

Nelson Claure

USA

Antonio Clavenna

Italy

Aldo Clerico

Italy

Marina Colombi

Italy

Peter Cooper

South Africa

Giuliana Cortese

Italy

Samuele Cortese

USA

Nicola Cotugno

Italy

Imelda Coyne

Ireland

Melanie Cree Green

USA

Noe Crespo

USA

Sarah Curtis

Canada

Estefania Custodio

Spain

Angela Czaja

USA

Dariusz Czaprowski

Poland

Fortunato D'Ancona

Italy

Jai Das

Pakistan

Daniel Datiko

Ethiopia

Andrew Day

Australia

Peter De Blank

USA

Vincenzo De Filippis

Italy

Elena De La Serna

Spain

Daniele De Luca

Italy

Maurizio De Martino

Kazakhstan

Jose Ricardo De Mello Brandao

Canada

Liliane De Swert

Belgium

Abbe De Vallejo

USA

Toity Deave

UK

Lidia Decembrino

Italy

Seyed Mohsen Dehghani

Iran

Joseph Delaney

USA

Clare Delany

Australia

Demet Demirkol

Turkey

Amanda Dempsey

USA

Eugene Dempsey

Ireland

Mark Deneau

Canada

Diana Di Gioia

Italy

Magnus Domellöf

Sweden

Marlos Domingues

Brazil

Olga Domínguez

Spain

Kirsty Donald

South Africa

Amelia Dos Santos

Brazil

Paulette Douek

Brazil

Amy Drendel

USA

Lizhong Du

China

Katrina Dubose

USA

Genevieve Dwyer

Australia

Alicja Dziuba

Poland

Christopher Bismarck Eke

Nigeria

Rajesh Elayedath

India

John Elliott

USA

Sahar El-Masry

Egypt

Matias Epifanio

Brazil

Leif Eriksson

Sweden

Günter Esser

Germany

Carlos Alfonso Fajardo

Canada

Fernanda Falcini

Italy

Alan Farrow-Gillespie

USA

Nancy Feeley

Canada

Emily Feinberg

USA

Jennifer Fenwick

Australia

David Fergusson

New Zealand

James Fett

USA

David Fields

USA

Guillermo Figueroa

Chile

Martijn Finken

Netherlands

Andrea Finocchi

Italy

Margaret Fisher

USA

Agata Fiumara

Italy

Itziar Flamarique

Spain

James Foy

USA

Alessandra Franco

USA

David Freedman

USA

Anne Fuhlbrigge

USA

Hideki Fujita

Japan

Ryuji Fukazawa

Japan

Raoul Furlano

Switzerland

Orazio Gabrielli

Italy

Luisa Galli

Italy

Tracey Galloway

Canada

Javier Ganame

Canada

Michael Ganz

USA

Gemma Garcia-Fructuoso

Spain

Julian Gardiner

UK

Sumit Gaur

India

Cindy Gauvreau

Canada

Ana Claudia Germani

Brazil

Sambuddha Ghosh

India

Vania Giacomet

Italy

Peter Gerard Gibson

Australia

Mercedes Gil-Campos

Spain

Francis Gilchrist

UK

Lynn Gillam

Australia

Maria Teresa Giner

Spain

John Giuliano

USA

Bamini Gopinath

Australia

Magdalena Górnicka

Poland

Daniela Goursand

Brazil

Stephen Graham

Australia

Liliane Grangeot-Keros

France

Ian Griffin

USA

Dorota Groffik

Poland

Veit Grote

Germany

Christoph Grüber

Germany

Paulo Guerra

Brazil

Fernando Guerrero-Romero

Mexico

Ursula Guillen

USA

Freedom Nkhululeko Gumedze

South Africa

Bernard Guyer

USA

Prakhar Gyanesh

India

Michele Hamm

Canada

Klaus Hamprecht

Germany

Rachel Han

China

Thor Willy Ruud Hansen

Norway

Flo Harrison

UK

Annelies Hartman

Netherlands

Kenneth Harttgen

Switzerland

Mohammad Hasan

Canada

Nihal Hatipoglu

Turkey

Kyla Hayford

Canada

Willem Helbing

Netherlands

Lena Hellström-Westas

Sweden

Ulf Helwig

Germany

Marc Hendricks

South Africa

Karen Hendricks-Munoz

USA

Lise Hestbaek

Denmark

Kristin Heumann

USA

Michael Hewson

New Zealand

David Hill

Australia

Marisa Hilliard

USA

Robert Hilliard

Canada

Susan Hillier

Australia

Stefanie Hinkle

USA

Valeria Hirschler

Argentina

Harriet Hiscock

Australia

Anders Hjern

Sweden

Andrew Hodge

Australia

Iva Hojak

Croatia

Jens-Christian Holm

Denmark

Kam-Lun Ellis Hon

Hong Kong

Alan Horn

South Africa

Rosemary Horne

Australia

Christiane Horwood

South Africa

Md Iqbal Hossain

Bangladesh

Jonathan Hourihane

Ireland

Laura Howe

UK

Evelyn Hsu

USA

Mei-Chih Huang

Taiwan

Yhu-Chering Huang

Taiwan

Roman Hudec

Slovakia

Angela Huertas

UK

Li Hui

China

Tony Hulse

UK

Ya Ching Hung

USA

Hayley Hutchings

UK

Machteld Hylkema

Netherlands

Roland Ibekwe

Nigeria

Wilson Igwe

Nigeria

Shigeo Iijima

Japan

Ifeanyi Ike

Nigeria

Moshe Ipp

Canada

Susan Ireland

Australia

Mary Irvine

USA

Jarmo Jääskeläinen

Finland

Narasimhan Jagannathan

USA

Etienne Javouhey

France

Ruth Jepson

UK

Mizu Jiang

China

Tracy Jirikowic

USA

Mary Ann Johnson

USA

Susan Johnson

USA

Celeste Johnston

Canada

Malia Jones

USA

Sophie Jones

Australia

Iolanda Jordan

Spain

Jocelyne Just

France

Satish Kalhan

USA

Robert Kane

Australia

G Kang

India

Andrew Kapetanakis

UK

Christos Karatzios

Canada

John Katsimpris

Greece

Astrida Kaugars

USA

Ilias Kavouras

USA

Abby Kazley

USA

Alison Kemp

UK

Han Cg Kemper

Netherlands

Jorien Kerstjens

Netherlands

Jothydev Kesavadev

India

Anat Kesler

Israel

Jennifer Kett

USA

Arif Khan

UK

Divya Khattar

India

Umakanth Khatwa

USA

Abdelmoneim Kheir

Sudan

Dennis Kim

USA

Mi Kyung Kim

South Korea

Jonathan King

USA

Nicholas Kiraly

Australia

Russell Kirby

USA

Roumen Kirov

Bulgaria

Niranjan Kissoon

Canada

Heidi Klakk

Denmark

Bonita Klein-Tasman

USA

Rebecca Knibb

UK

Anders Koch

Denmark

Ozlem Koksal

Turkey

Michael Kramer

Canada

Baruch Krauss

USA

Guha Krishnaswamy

USA

Claudia Elisabeth Kuehni

Switzerland

Hemant Kulkarni

USA

Chandrakanta Kumar

India

Rashmi Kumar

India

Sunil Kumar

India

Ayca Esra Kuybulu

Turkey

Harrie Lafeber

Netherlands

Chandrakant Lahariya

India

Abhimanu Lall

UK

Dionísia Lamônica

Brazil

Amy Lampard

USA

Claudio F. Lanata

Peru

Lara Latimer

USA

Elise Launay

France

Luisa Lazaro

Spain

Robin Leboeuf

USA

Burton Lee

USA

Hung-Chang Lee

Taiwan

Nichung Lee

Taiwan

Brigitte Lemyre

Canada

Afolabi Lesi

Nigeria

Danielle Levac

Canada

Joyce Li

USA

Yanhong Li

China

Chien-Chang Liao

Taiwan

Selma Liberato

Australia

Chung-Ying Lin

Taiwan

Ornella Lincetto

Italy

Linda Lindeke

USA

Alexandre Linhares

Brazil

Peter Lio

USA

Tanya Little

Australia

Rong Liu

China

Youxue Liu

China

Clifford Lo

USA

Robert Locke

USA

Donald Lollar

USA

Eric Loucks

USA

Jean Lowe

USA

R.Brian Lowry

Canada

Olinda Luiz

Brazil

Michele Maddux

USA

K S Madhusudhan

India

Ana Maria Madrid

Chile

Everett Magann

USA

Akhil Maheshwari

USA

Ashwini Mallappa

USA

Caterina Mammina

Italy

Sheetal Manicklal

South Africa

Cedric Manlhiot

Canada

Weerawat Manosuthi

Thailand

Beatriz Marciano

USA

M. Loredana Marcovecchio

Italy

Kevin Marks

USA

Pedro Marques-Vidal

Switzerland

Silvia Martínez Valverde

Mexico

Saidur Mashreky

Bangladesh

Kabir Masokano

Nigeria

Wilbert Mason

USA

Amit Mathur

USA

Mogomotsi Matshaba

Botswana

Ines Mattos

Brazil

Gerardo Maupome

USA

Roch Listz Maurice

Canada

Philip May

USA

Dorothy Mbori-Ngacha

South Africa

Elizabeth Mcclure

USA

Nichola Mccullough

UK

Jenny Mcdonald

Australia

Andrew Mcfarlane

Canada

Brian Mcfarlin

USA

Meghan Mcgrady

USA

Philip Mcternan

UK

Larissa Mendes

Brazil

Gregorio Paolo Milani

Italy

Jeremy Miles

USA

Annarita Milone

Italy

Jonathan Mintzer

USA

Neeti Mittal

India

Omer Moghraby

UK

Yasser Moharram

Kuwait

Anup Mohta

India

Olugbenga Mokuolu

Nigeria

Diego Moliner-Urdiales

Spain

Paula M.C. Mommersteeg

Netherlands

Maureen Monaghan

USA

Michel Mondain

France

Marco Montevecchi

Italy

Giovanni Montini

Italy

Kotsedi Daniel Monyeki

South Africa

Francisco J Morales-Olivas

Spain

Allisyn Moran

USA

Nathalie Morel

France

Kevin Morris

UK

Brian Moseley

USA

Steven Moss

Canada

Dezhi Mu

China

Mariya Mukhtar Yola

Nigeria

Paul Mullan

USA

Luke Mullany

USA

Essey Kebede Muluneh

Ethiopia

Caroline Mulvaney

UK

Brendan Murphy

Ireland

Tasha Murphy

USA

Victoria Nakibuuka

Uganda

Estelle Naumburg

Sweden

Patti-Jean Naylor

Canada

John Neff

USA

Ellen Nelissen

Tanzania

Anne Nesbitt

Sierra Leone

Tryggve Nevéus

Sweden

Karen New

Australia

Anna Newton

USA

Signe Smith Nielsen

Denmark

Christopher Niemiec

USA

Andreas Nilsson

Sweden

Fidelis Njokanma

Nigeria

Melanie Noel

USA

Elio Novembre

Italy

Jane Noyes

UK

Paolo Nucci

Italy

Ulugbek Nurmatov

UK

James Nuttall

South Africa

Emeka Nwolisa

Nigeria

Ikechukwu Obi

Nigeria

Louise O'Brien

USA

Fola Odetola

USA

Tagbo Oguonu

Nigeria

April Oh

USA

Bernadette O'Hare

Malawi

Ngozi Ojinnaka

Nigeria

Henrietta Uchenna Okafor

Nigeria

Bolajoko O. Olusanya

Nigeria

Jennifer O'Neill

USA

Nnamdi Benson Onyire

Nigeria

Rianne Oostenbrink

Netherlands

Aybars Ozkan

Turkey

Fabio Paglialonga

Italy

Elijah Paintsil

USA

Greta Palmer

Australia

Sabela pdela

Spain

Karthikeyan pnthaman

UK

Prabhu Parimi

USA

Dusan Paripovic

Serbia

Jae Park

USA

Kathryn Parkinson

UK

Sameer Patel

USA

Nivedita Patni

USA

Maria Francesca Patria

Italy

Ashok Patwari

India

Massimiliano Pau

Italy

David Paul

USA

Stephen Pearlman

USA

Freya Pearson

UK

Cecile Alexandra Peltier

France

Alexia Pena

Australia

Paul Perez

France

James Perrin

USA

Cynthia Peterson

Switzerland

William Pickett

Canada

Caroline Pickstone

UK

Olivier Picone

France

Jan Patricia Piek

Australia

Joseph Pierre

USA

Russell Pierre

Jamaica

Terri Pikora

Australia

Martin Pinquart

Germany

Andreia Nogueira Pizarro

Portugal

Sandra Plachta-Danielzik

Germany

Ujjal Poddar

India

Marko Pokorn

Slovenia

Kinga Polanska

Poland

Henry Potts

UK

Wagner Prado

Brazil

Naomi Priest

Australia

Robert Primhak

UK

Jardena Puder

Switzerland

Olga Puig

Spain

Jose M. Quintana

Spain

Susan Racette

USA

Aiman Rahmani

United Arab Emirates

Shaman Rajindrajith

Sri Lanka

S.V. Rana

India

Shripada Rao

Australia

Sabrina Rasheed

Bangladesh

Iris Rathwell

UK

Niamh Redmond

UK

Stéphane A. Régnier

Switzerland

Gregor Reid

Canada

Susan Reid

Australia

Leslie Rescorla

USA

Gitta Reuner

Germany

Corsino Rey

Spain

Dong-Wook Rha

South Korea

Roberto Régis Ribeiro

Brazil

Jacques Rigo

Belgium

Ana Rito

Portugal

Emmanouil Rizos

Greece

Christine Roberts

USA

Stephen Robertson

New Zealand

Joan Robinson

Canada

Andrea Ronchi

Italy

Anna Rosati

Italy

Sarah Rose

Canada

Rebecca Rosenberg

USA

Liza Rovniak

USA

Sabine Roza

Netherlands

Franca Rusconi

Italy

Fiona Russell

Australia

Tzang Ruu-Fen

Namibia

Olli Ruuskanen

Finland

Sandra Saldivia

Chile

Cassandra Salgado

USA

Shamiel Salie

South Africa

Helen Sammons

UK

Luciana Maria Malosa Sampaio

Brazil

Megan Sandel

Canada

Leopoldo Santos-Argumedo

Mexico

Grammati Sarri

UK

Pieter Sauer

Netherlands

Laura Sauve

Canada

Reg Sauve

Canada

Gregory Sawicki

USA

Sadath Sayeed

USA

Angelika Schlarb

Germany

Lourdes Schnaas

Mexico

Clive Schneider-Friedman

Canada

Edgar Schoen

USA

Stephen Scholand

USA

Lode Schuerman

Belgium

Chris Scott

South Africa

Rosie Scuccimarri

Canada

Michael Seear

Canada

Ruud Selles

Netherlands

Fred Semitala

Uganda

Katherine Semrau

USA

Ali Sepahdari

USA

Divyen Shah

UK

Prakeshkumar Shah

Canada

Gabriel Shaibi

USA

Preeti Shanbag

India

Christina Shay

USA

Ross Shegog

USA

Xiaoyang Sheng

China

Julian Shield

UK

Linda Shields

Australia

Marwan Shinawi

USA

Shimon Shiri

Israel

Kevin Short

USA

Mohamed Shoukri

Canada

Andrew Sidebottom

UK

Hermundur Sigmundsson

Norway

Kathleen Sim

UK

Rune J Simeonsson

USA

Peter Simm

Australia

Burkhard Simma

Austria

Ana Cristina Simoes E Silva

Brazil

Jaivir Singh

India

Tina Slusher

Nigeria

Lucy Smith

UK

Henry Smithson

UK

Wei-Tsuen Soong

Taiwan

Michael Spear

USA

Kaye Spence

Australia

Zoltan Spolarics

USA

Jose Spolidoro

Brazil

Chandrashekhar Sreeramareddy

Nepal

Neeraj Mohan Srivastava

India

Thorsten Stanley

New Zealand

Lindsay Stead

UK

Philippe Steenhout

Switzerland

Olle Stendahl

Sweden

Christopher Stevenson

Australia

Parmi Suchdev

USA

Gautham Suresh

USA

Jc Suris

Switzerland

Kohta Suzuki

Japan

Ehsan Syed

USA

H. Szajewska

Poland

Beckie Tagbo

Nigeria

Mohammed Taha

Ethiopia

Tomoyuki Takano

Japan

Hiroshi Tanaka

Japan

Neelam Taneja

India

Phillip Tarr

USA

Tülay Tarsuslu Simsek

Turkey

Arianne Teeuw

Netherlands

Jonathan Teitelbaum

USA

Luigi Terracciano

Italy

Elaine Tham

Australia

Irén Tiberg

Sweden

Peter Tilley

Canada

Gabriel Tobón

Colombia

Robert Tolan

USA

Sonia Toni

Italy

Karina Top

Canada

Alberto Tozzi

Italy

Dat Tran

Canada

Leonardo Trasande

USA

Andrea Trembath

USA

Victoria Trenchs

Spain

Jeanie Tryggestad

USA

David Tudehope

Australia

Michael Tunik

USA

Simona Turcu

UK

Paul Turner

UK

Deborah Tuttle

USA

Agozie Ubesie

Nigeria

Ekrem Unal

Turkey

Zsolt Urban

USA

Fabio Uxa

Italy

Pietro Vajro

Italy

Hugo Van Bever

Singapore

Agnes Van Den Hoogen

Netherlands

Marietta Van Der Linden

UK

Ruurd M. Van Elburg

Netherlands

A.M. Van Furth

Netherlands

Jens Vanselow

Germany

George Vaos

Greece

Carina Venter

UK

Giovanni Vento

Italy

Walter Verbrugghe

Belgium

Massimiliano Veroux

Italy

M. Cristina Victorio

USA

Maria Marluce Vilela

Brazil

Carolina Villegas Alvarez

Mexico

Christina Vinter

Denmark

Dan Waisman

Israel

Peter Waiswa

Uganda

Anne Walsh

Australia

Robinson Wammanda

Nigeria

Gustavo Wandalsen

Brazil

Guoying Wang

USA

Frances Ward

USA

Anna Wasilewska

Poland

Peter Watkin

UK

Wade Watson

Canada

Michel Weijerman

Netherlands

Emma Weisblatt

UK

Zita Weise Prinzo

Switzerland

Ewa Wender-Ozegowska

Poland

Keith West

USA

William Whitehead

USA

Julio Wiederkehr

Brazil

Barbara Willey

UK

Connie Williams

Canada

Paige Williams

USA

Noreen Willows

Canada

Esko Wiltshire

New Zealand

Corinna Winter

Germany

Ann-Britt Wiréhn

Sweden

Stefan Wirth

Germany

Eric Wobudeya

Uganda

Andrea Wolfler

Italy

Lisa Wood

Australia

Anne Wormsbecker

Canada

Charlotte Wright

UK

Courtney Wusthoff

USA

Aparecida Yulie Yamamoto

Brazil

Tiebin Yan

China

Tao Yang

China

Yee Ian Yik

Malaysia

Bridget Young

UK

Mary Young

USA

Syed Wamique Yusuf

USA

Nicola Zampieri

Italy

Nikolay Zavadenko

Russian Federation

Phyllis Zelkowitz

Canada

Huan Zeng

China

Zhiguang Zhou

China

Joseph Zickafoose

USA

Stefan Zielen

Germany

Vibeke Zoffmann

Denmark

Rose Zulliger

USA

## Pre-publication history

The pre-publication history for this paper can be accessed here:

http://www.biomedcentral.com/1471-2431/14/7/prepub

